# Look Alike, Sound Alike: Phenocopies in Steroid-Resistant Nephrotic Syndrome

**DOI:** 10.3390/ijerph17228363

**Published:** 2020-11-12

**Authors:** Francesca Becherucci, Samuela Landini, Luigi Cirillo, Benedetta Mazzinghi, Paola Romagnani

**Affiliations:** 1 Pediatric Nephrology and Dialysis Unit, Meyer Children’s Hospital, Viale Pieraccini 24, 50139 Florence, Italy; luigi.cirillo@meyer.it (L.C.); benedetta.mazzinghi@meyer.it (B.M.); paola.romagnani@unifi.it (P.R.); 2 Department of Biomedical, Experimental and Clinical Science “Mario Serio”, University of Florence, Viale Morgagni 50, 50134 Florence, Italy; samuela.landini@unifi.it

**Keywords:** phenocopies, whole-exome sequencing, steroid-resistant nephrotic syndrome, genetics

## Abstract

Steroid-resistant nephrotic syndrome (SRNS) is a clinical picture defined by the lack of response to standard steroid treatment, frequently progressing toward end-stage kidney disease. The genetic basis of SRNS has been thoroughly explored since the end of the 1990s and especially with the advent of next-generation sequencing. Genetic forms represent about 30% of cases of SRNS. However, recent evidence supports the hypothesis that “phenocopies” could account for a non-negligible fraction of SRNS patients who are currently classified as non-genetic, paving the way for a more comprehensive understanding of the genetic background of the disease. The identification of phenocopies is mandatory in order to provide patients with appropriate clinical management and to inform therapy. Extended genetic testing including phenocopy genes, coupled with reverse phenotyping, is recommended for all young patients with SRNS to avoid unnecessary and potentially harmful diagnostic procedures and treatment, and for the reclassification of the disease. The aim of this work is to review the main steps of the evolution of genetic testing in SRNS, demonstrating how a paradigm shifting from “forward” to “reverse” genetics could significantly improve the identification of the molecular mechanisms of the disease, as well as the overall clinical management of affected patients.

## 1. Introduction

Nephrotic syndrome (NS) is a clinical picture common to different glomerular and non-glomerular diseases, with variable response to treatments and heterogeneous outcomes. In children and adolescents, primary NS is classified as steroid-sensitive (SSNS) and steroid-resistant NS (SRNS) based on the response to standard steroid treatment. Patients with SRNS have a significantly worse prognosis due to an increased risk of developing chronic kidney disease (CKD) and severe side effects of immunosuppressive therapies that are commonly used as second-line treatments.

Genetic testing has become a valuable diagnostic tool in defining the etiology of SRNS, leading to the identification of a genetic cause in about 30% of patients (monogenic podocytopathies). Genetic SRNS has no chance to respond to immunosuppressive therapies and frequently progresses to end-stage kidney disease (ESKD), accounting for about 15% of pediatric cases [1,2]. The identification of genetic SRNS is pivotal in order to tailor therapeutic measures and prognosis prediction, as well as genetic counseling for reproductive and organ transplantation purposes.

The advent and spreading of next-generation sequencing (NGS) have significantly improved diagnostic strategies for inherited diseases including SRNS. Indeed, simultaneous sequencing of multiple genes has become feasible, time- and cost-effective and is increasingly performed, either in diagnostics or in research. However, after an initial phase, widening the number of genes analyzed by NGS in patients with SRNS did not result in a significant improvement in the diagnostic rate of genetic forms that stabilized around 30%, thus hindering the advent of precision medicine in SRNS. Recent evidence supports the hypothesis that “phenocopies” could account for a non-negligible fraction of SRNS patients who are currently classified as non-genetic upon standard genetic testing (i.e., genetic analysis limited to monogenic podocytopathies) [3,4], paving the way for a more comprehensive understanding of the overall genetic background of each single patient and for the elucidation of potential genetic biomarkers of disease progression in SRNS. Following its first introduction in medicine, the term phenocopies is currently used to identify patients sharing the same phenotype who are carriers of different genotypes. Genetic testing is crucial for detecting phenocopies. However, it can be insufficient.

The aim of this work is to review the main steps of the evolution of genetic testing in SRNS, demonstrating how a paradigm shifting from “forward” to “reverse” genetics could significantly improve the identification of the molecular mechanisms of the disease, as well as the overall clinical management of affected patients. We will also review the concept of phenocopies in medicine in order to provide evidence that inherited nephropathies caused by mutations in other than canonic podocyte genes but presenting as isolated SRNS can be defined as phenocopies of monogenic podocytopathies. In this view, “reverse” genetics, an approach to identifying the effect on phenotype of a specific genotype, represents one of the potential strategies for their identification.

## 2. The Genetic Revolution: From Single Gene Analysis to Extended Genotyping

The completion of the “Human Genome Project” in 2001 initiated a rapid expansion of knowledge about the human genome, allowing accurate assessment of its sequence, topography and extent of variation.

Sanger sequencing, referred to as “first-generation” sequencing, has represented the gold standard method of DNA sequencing for more than 25 years since many Mendelian diseases are caused by mutations in a single disease-causing gene [5]. The clinical use of Sanger sequencing is usually restricted to the testing of single genes one at a time, typically at a cost of USD 500 *per* Megabase (Mb) [6]. Therefore, Sanger sequencing requires a high clinical suspicion of a specific diagnosis (hypothesis-driven sequencing) and is usually initiated by geneticists.

During recent decades, the development of “second-generation” sequencing technology (also referred to as NGS) resulted in a wide application of genomic analysis not only in research, but also in clinical settings. NGS is based on massive parallel sequencing, that is to say the simultaneous analysis of multiple genes in a single run. They can include a selection of genes of interest (targeted panels), all coding DNA regions (whole-exome sequencing, WES) or the entire genome (whole-genome sequencing, WGS).

The technologic strengthening of sequencing platforms coupled to the implementation of bioinformatics pipelines to manage and analyze large genomic datasets, together with the progressive decrease in sequencing costs, has been making NGS a hypothesis-free, highly informative tool in exploring the molecular causes of Mendelian disorders and understanding the mechanisms underlying their pathogenesis [7]. As a consequence, genetic testing has started to become a tool managed also by clinicians, even though this shift is still ongoing.

Targeted gene panels are widely used in clinical diagnostics to identify genetic causes for disease groups involving several causative genes. However, gene panels are rapidly outdated as new disease genes continue to be discovered, needing frequent re-design. Indeed, since 2010, new genes are identified at a rate of approximately three genes *per* week [8] and one every 28 exomes performed [9] and many laboratories turned to WES as an even more efficient approach to genetic diagnostics of inherited diseases. Currently the cost for WES is ~ USD 1000 *per* patient, and results are usually provided in about 10 weeks. This approach allows clinicians to expand the genetic spectrum of diseases and then to initiate future reanalysis as new genes are discovered, without resampling the patient [10,11]. Of note, the time and economic costs of genetic testing have shifted from sequencing *per se* to diagnostic interpretation, that nowadays represent the major barrier for genetic testing to be routinely implemented in daily clinical practice [12,13]. Interpretation of WES sequence data, with particular regard to the findings of variants with uncertain significance (VUS) and unexpected genetic results, makes the diagnosis process extremely complicated and time-consuming. As new genes are discovered, clinicians and geneticists have to face the additional challenge of continuously assessing the strength of genotype–phenotype associations. Indeed, by providing increasing amounts of data, clinical research either purposes new correlations between gene/genes and phenotype or raises doubts on those previously reported [14]. Therefore, VUS probably represent the main strength and the main limit of extended genotyping.

Genomic medicine has been significantly implemented in nephrology. Inherited nephropathies represent the cause of approximately 10% of adult ESKD [15,16] and about two thirds of pediatric nephropathies [17,18]. As a consequence, genetic testing is increasingly used to assess the human genome for causal variants for a variety of kidney disorders, including SRNS.

**Figure 1:**
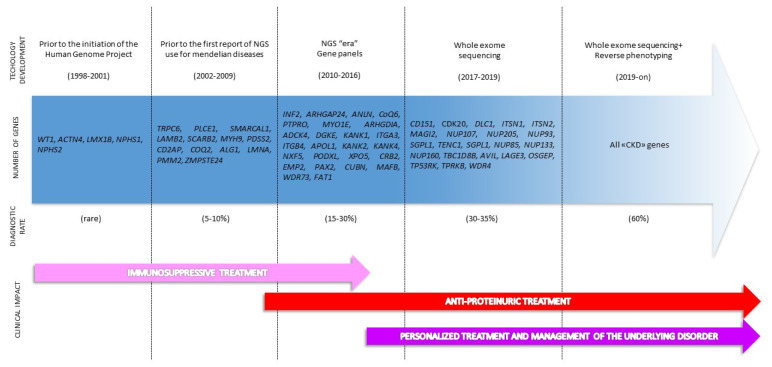
Evolution of genetic diagnosis in steroid-resistant nephrotic syndrome (SRNS). This picture illustrates the advances in the genetic diagnosis of SRNS encompassing technological improvement and analytic strategies. The main cornerstones of sequencing technology development, the genes reported as causative of SRNS and the diagnostic rates are reported. NGS, next-generation sequencing; CKD, chronic kidney disease.

The discovery of the first gene responsible for SRNS dates back to 1998, when positional cloning identified mutations in *NPHS1* in neonates with congenital NS of the Finnish type [19] (Figure 1). In the last three decades, the number of genes potentially causative for SRNS has continuously increased [17,20] (Figure 1). Currently, in the Online Mendelian Inheritance in Man (OMIM) database [21], about 30 genes are listed under the category of NS or focal segmental glomerulosclerosis (FSGS). Strikingly, all these genes are expressed by podocytes, the culprit cells of glomerular filtration barrier integrity. According to their function, these genes are usually referred to as podocytopathy genes and the diseases caused by their mutations as monogenic podocytopathies [22] (Figure 1). In addition, many other genes (about 34) are listed as causative for syndromic forms of SRNS or FSGS (e.g., Alport syndrome, nail–patella syndrome) that are usually clinically recognized with appropriate diagnostic work-up.

In the 21st century, NGS led to a marked increase in the diagnostic rate of genetic forms of SRNS (approximately doubled in comparison to traditional Sanger sequencing), especially in children and adolescents and in familial cases [23,24,25,26,27,28,29,30]. However, NGS significantly improved our ability to identify a genetic cause also in isolated sporadic SRNS [31], so genetic testing has been widely performed with the aim of informing therapies, avoiding unnecessary use of immunosuppressive treatment whenever indicated [29,32]. Notwithstanding the increase in the diagnostic power of genetic testing performed with NGS and despite the application in large cohorts of patients [26], the diagnostic rate of genetic forms of SRNS did not significantly improve over 30% even after an impressive increase in the number of podocytopathy genes analyzed by NGS (gene panels and WES) (Figure 1). Of note, some patients who are currently classified as non-genetic SRNS do not respond to immunosuppressive treatment and anyway progress to ESKD, claiming for a genetic cause. These patients often face a “diagnostic odyssey”, which includes multiple misdiagnoses, unnecessary tests, and unspecific treatment.

Given the lack of specificity of clinical features of many forms of CKD, an extended genetic testing including all the genes known to cause CKD may represent a strategy to improve the diagnostic rate even in SRNS, thus helping to avoid the “diagnostic odyssey” that patients often face [33] (Figure 1). As previously stated, such an extended strategy risks to run into unexpected genetic findings, such as potentially pathogenic variants in genes not reported as causative of SRNS. Additional efforts and algorithms, including the use of reverse phenotyping, must be put in place in order to assess the pathogenicity of these genetic findings. According to the literature, these patients could be finally named “phenocopies”.

## 3. What Is a Phenocopy?

The concept of “phenocopy” was first introduced in 1935 by Goldschmidt in describing different types of phenotypic abnormalities induced by heat and chemical compounds in Drosophila’s larvae. He speculated that non-genomic modifications (i.e., modifications of experimental conditions such as hypoxia, heat, chemicals) could have the same effect on phenotype as genetic mutations, thus resulting in indistinguishable clinical pictures copying the phenotype of mutants [34]. In 1958, Buchner pointed out that genetic abnormalities could be copied by exogenous insults, resulting in similar clinical phenotypes, concluding that “exogenous malformations are in principle phenocopies of malformations which may also be genetic”. As a consequence, the first concept of phenocopy referred to variations in phenotype caused by environmental, non-hereditary conditions, identical to the genotype-determined phenotype of another individual [35]. However, studies performed on invertebrates provided evidence that environmental stressors could act by modifying the mechanism of action of proteins and enzymes produced by genetic variants only under specific conditions. In this view, the phenotypic effects of specific genes, when these are only apparent under certain environmental conditions and identical to the usual phenotypic effects of other possibly isoallelic genes, were referred to as phenocopies [34].

Following these first reports, a long-lasting debate on whether the term phenocopy should be used to refer to a purely environmentally induced phenotype started over, especially for human diseases.

Indeed, the term phenocopy was introduced in human medicine in the early 1950s [36], but its use has been vague and frequently conflicting. Phenocopies of genetic disorders have been described as the consequence of environmental exposure to drugs, toxics, vitamins deficiency or infective agents [37]. As an example, endemic cretinism due to iodine deficiency mimics the phenotype of autosomal recessive (AR) Pendred’s syndrome [34]. Similarly, thalidomide embryopathy comprises a great variety of skeletal and organ malformations that closely mimic genetic disorders (e.g., Holt–Oram syndrome, radius aplasia-thrombocytopenia syndrome), thus representing their phenocopies [34,38].

In this view, environmental stimuli are considered sufficient to determine the onset of specific clinical traits (phenotype). However, one can speculate that external agents act by modifying the expression of environment-responsive genes, as described in invertebrates and animal models [34]. The gene–environment interaction leading to the appearance of specific phenotypic traits partially questions the original concept of phenocopy, opening more complex mechanisms of gene expression and phenotypes determination. As a consequence, the concept of the phenocopy of human diseases has been recently expanded.

**Figure 2:**
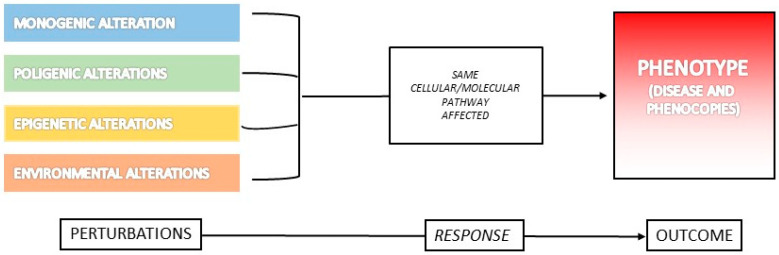
Mechanisms of disease convergence and generation of phenocopies. This picture illustrates the mechanisms responsible for the onset of overlapping clinical phenotypes, namely phenocopies. Monogenic, polygenic, epigenetic and environmental alterations can result in similar phenotypic traits, probably converging on the same functional pathway.

In support of this interpretation, disease convergence is the process by which diseases of different root causes share the same (or extremely similar) phenotype. Notwithstanding their sophistication, complex biological systems, such as vertebrates and humans, tend to respond to different perturbations with similar patterns that are finite and repeatable [39]. In other words, cellular perturbations converge to a limited set of outcomes, regardless of their causes (e.g., genetic, toxic) (Figure 2). In this view, not only monogenic (i.e., genetic mutations in a single gene, causing a large size effect, necessary and sufficient to determine the clinical picture) and environmental, but also polygenic (i.e., mutations in more than one gene, each with a small or even unnoticeable size effect) and epigenetic alterations can all converge to the same phenotype. This is probably due to the effect on the same functional pathway that can be affected by different types of abnormal stimuli (Figure 2). Accordingly, all of them can “phenocopy” the corresponding genetic disease.

Indeed, a phenocopy can be defined as a phenotypic trait or disease that resembles the trait expressed by a particular genotype, but in an individual who is not a carrier of that genotype [40]. This can conceptually mean that mutations in genes different from those classically reported in association with a specific phenotype could result in similar phenotypic effects, thus being completely indistinguishable.

In addition, the number of genes whose mutations are capable of causing the onset of a clinical phenotype (normal or abnormal) not only depends on understanding the molecular roots that drive the observed outcome, but also on our technical ability to search for them when performing genetic sequencing. Indeed, by increasing our understanding of pathophysiological mechanisms, the number of genes associated with a disorder strikingly increases (locus heterogeneity). These genes are usually part of the same protein complex, organelle or functional subunit of the cell, cooperating in maintaining the functional integrity of a cellular and/or metabolic pathway [39]. On the other hand, by expanding the number of genes that can be simultaneously analyzed in an individual in a single run of sequencing (NGS), the association between a phenotypic trait and previously unrecognized genetic loci is commonplace in the genomic era, making the definition of phenocopy challenging. Indeed, “not being carrier of a genotype” can be simply the consequence of not having searched deep enough or not having previously associated a phenotypic trait to a genetic locus. In a more inclusive view, diseases caused by mutations in different genes resulting in similar, although not identical, clinical phenotypes can be considered as phenocopies.

### Examples of Phenocopies in Human Diseases

The term phenocopy is widely used in cardiology, ophthalmology, neurology, immunology and oncology. However, its use is frequently inconsistent or confusing, not only in different fields of medicine, but also within the same discipline (Table 1). Here, we provide some examples of the use of the term phenocopy in different fields of medicine.

Together with endemic cretinism and thalidomide embryopathy (see above), congenital toxoplasmosis represents an example of a purely environmental-acquired disease (phenocopy) mimicking a rare disorder, namely grade 3 North Carolina macular dystrophy (NCMD), that is caused by mutations in the MCDR1 locus on chromosome 6 or in the MCDR3 locus on chromosome 5 [41]. However, foveal hypoplasia and torpedo maculopathy are commonly referred to as phenocopies of grade 1 and grade 2 NCMD, but their pathogenic mechanisms are still under investigation [41]. Whatever the abnormal stimuli (either environmental or unknown), the term “phenocopy” is commonly used by ophthalmologists to refer to these conditions.

However, this term has a far-reaching complexity. The molecular basis of inherited disorders like hypertrophic cardiomyopathy is quite clearly identified. Mutations in sarcomeric genes account for about 60% of cases of hypertrophic cardiomyopathy [42,43]. Notwithstanding this, a genetic alteration in sarcomeric genes is lacking in up to one third of patients, so that the diagnosis of hypertrophic cardiomyopathy remains clinical rather than genetic [44]. Interestingly, an additional 10–15% of patients are affected by systemic diseases referred to as phenocopies of hypertrophic cardiomyopathy. Some of these disorders (such as Fabry disease, MELAS or PRKAG2 cardiomyopathy) have defined genetic bases, while others (such as AL amyloidosis) are caused by different, not fully elucidated, mechanisms [43,44]. In this example, a patient can be classified as a phenocopy if they are either affected by a genetic disease with a different genetic basis or if the pathophysiologic mechanism is yet to be clarified. As a consequence, a phenocopy of hypertrophic cardiomyopathy can be represented by systemic diseases that are genetic in nature or by disorders caused by complex, probably multi-factorial and still unknown, mechanisms.

In addition, a significant fraction of primary immunodeficiencies are monogenic inherited diseases, with more than 350 single-gene defects identified [45]. Immunodeficiencies caused by somatic mutations or autoantibodies are defined as phenocopies of primary immunodeficiencies [46]. As an example, autoimmune lymphoproliferative syndrome (ALPS) is characterized by defective Fas-mediated apoptosis in lymphocytes caused by genetically impaired regulation of Fas, Fas ligand and their effectors [47]. However, a proportion of patients with a clinical phenotype suggestive of ALPS do not show mutations in ALPS genes. Nowadays, somatic mutations in the *FAS* gene are recognized as the second most common cause of ALPS [47,48]. Interestingly, somatic mutations in the *FAS* gene have also been reported to be responsible for ALPS in patients with haploinsufficient germline heterozygous *FAS* mutations, probably acting as a second genetic hit [49]. Similarly, somatic mutations in the *NLRP3* gene may account for a large majority of patients (up to 70%) affected by cryopyrinopathies, a group of AD diseases caused by germline mutations in the *NLRP3* gene [47]. The most characterized example of phenocopies of primary immunodeficiency due to autoantibodies is probably represented by acquired angioedema, a disease caused by autoantibodies against C1-inhibitor clinically mimicking the genetic disorder arising from mutations in the *SERPING1* gene encoding for C1-inhibitor [50]. Similarly, autoantibodies against IL-17 and IL-22 can result in clinical pictures indistinguishable from chronic mucocutaneous candidiasis, an AR disease caused by mutations in genes encoding cytokines, such as IL-17 and IL-22, involved in immune response against fungal infections [51], whereas autoantibodies against IFN-γ can result in clinical manifestations (e.g., opportunistic infections) similar to those seen in patients who have genetic defects in the IL-12/IFN-γ axis. Phenocopies of primary immunodeficiencies caused by somatic mutations or autoantibodies usually occur later in life and the clinical phenotype can be milder.

As an additional level of sophistication, the term phenocopy is also used to indicate patients showing clinical features suspected of a monogenic disorder who anyway test negative to genetic screening. Parkinson’s disease shows a clear Mendelian inheritance pattern and familial aggregation in about 3–10% of cases [52]. More than 20 disease-segregating genes or loci causing Parkinson’s disease have been identified so far, with *SNCA*, *LRRK2*, Parkin and *PINK1* representing the most frequently involved [53,54]. However, inheritance patterns do not always follow classic Mendelian genetics and incomplete penetrance and variable disease expressivity are common among affected family members of a pedigree. Thus, genetic testing turns out to have negative results in a significant number of affected individuals belonging to the same family. These patients are called phenocopies of Parkinson’s disease. In the majority of phenocopies, the cause of Parkinson’s disease could not be identified. Similarly, breast cancer frequently shows familial aggregation, with disease affecting many members of the family. Approximately 30% of this hereditary cancer risk is explained by genetic variants in high-penetrance susceptibility genes such as *BRCA1* or *BRCA2* [55,56]. However, some women belonging to families with an identified pathogenic variant in *BRCA1* or *BRCA2* will develop cancer despite testing negative for the family’s pathogenic variants and are often denoted as phenocopies [56,57]. Recent studies provided evidence that phenocopies occur more frequently than expected by chance, leading to the hypothesis that these families may have pathogenic variants in additional genes, which can be capable of causing cancer in the absence of alterations in *BRCA* genes [56].

In some cases, a phenocopy is considered a purely genetic alternative diagnosis to the main clinical hypothesis. As an example, Huntington disease (HD) is one of the best examples of genetic disorders caused by triplet expansion in the *HTT* gene. However, not all patients with an HD phenotype carry the pathological expansion in *HTT*, and the positive diagnostic rate of genetic testing is poor [58]. Some inherited disorders have historically been recognized as manifesting or evolving as HD-like phenotypes and referred to as HD phenocopies. HD phenocopies account for about 1% of suspected HD cases [58,59]. Of note, all recognized HD phenocopies have a genetic etiology and environmental causes of the HD phenotype have not been described.

As a final consideration, an additional conflict in defining what is a phenocopy comes from cardiology. Indeed, Brugada phenocopies are defined as clinical entities sharing the typical ECG pattern of Brugada syndrome in the absence of congenital Brugada syndrome [60]. Brugada phenocopies are transitory in nature, resolving by eliminating the trigger. Although specific conditions have been reported as Brugada phenocopies (e.g., metabolic abnormalities, ischemia, mechanic compression, myocardial and pericardial diseases), their molecular mechanisms still remain unidentified, making the differential diagnosis between Brugada syndrome and Brugada phenocopies extremely challenging [61]. Moreover, the pathogenic mechanisms of congenital Brugada syndrome itself are still purely understood, since mutations in *SCN5A* account for no more than 20–30% of cases. The molecular cause of the remaining cases is unknown [62].

## 4. From Phenotype to Genotype and Backwards in SRNS

**Figure 3:**
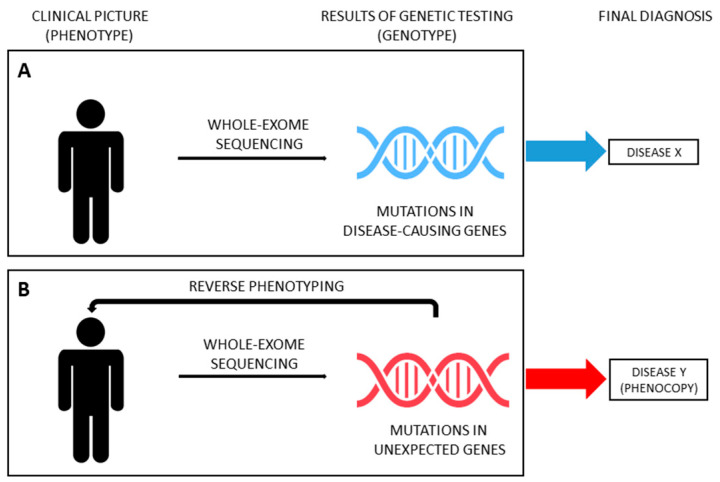
From phenotype to genotype, and back. (**A**) Genetic testing can be used to confirm the clinical suspect of a genetic disease, looking for mutations in disease-causing genes (from phenotype to genotype). Genetic testing can be performed either with traditional Sanger sequencing (if only one gene can cause the phenotype or if the clinical suspect is extremely high) or with NGS (if more than one gene is known to cause the disease). (**B**) Extended genotyping can result in unexpected genetic findings (i.e., mutations in genes not classically reported in association with the clinical phenotype of the patient). Reverse phenotyping represents a strategy to verify the hypothesis of a specific genetic diagnosis (from genotype to phenotype). By looking for subtle, previously overlooked clinical signs or symptoms related to the genetic diagnosis, reverse phenotyping can reclassify the diagnosis and initiate personalized clinical management.

Thanks to the improvements and cost-cut of massive parallel sequencing techniques, in recent years, several studies have been carried out using NGS in large cohorts of patients affected by kidney diseases, paving the way for its use in the standard clinical work-up, when genetic etiology is suspected. Genetic testing allows clinicians to test the hypothesis that a phenotype is caused by mutations in disease-causing genes (moving from phenotype to genotype, Figure 3A). The continuous growth in the number of genes reported in association with a specific disease (phenotype) makes NGS ideal to address this diagnostic issue (genotype). However, the spreading use of NGS forces geneticists and clinicians to deal with the problem of unexpected results (i.e., mutations in genes not previously reported in association with the phenotype) and with the challenge of disease reclassification, especially with WES. WES has proven to be useful for the reclassification of various inherited nephropathies, such as nephronophthisis, ciliopathies and CAKUT [63,64,65,66,67,68,69,70,71]. Strikingly, in recent years, WES has been successfully applied also in unselected cohorts of adult patients with ESKD of unknown origin, identifying a monogenic cause in nearly 10% of cases who were therefore reclassified [72,73]. As above mentioned, patients presenting with a phenotype corresponding to a specific hereditary disease but not carrying the expected genotype can be considered phenocopies. However, establishing a causative relationship between (unexpected) genotype and phenotype (moving from genotype to phenotype, also known as reverse genetics) could be tiresome. Very recently, the genetics of SRNS started this challenge.

Genetic testing with NGS has become a reliable and affordable diagnostic tool in patients with early-onset idiopathic SRNS, leading to an improvement in the personalized management of about 30% of cases who result as being affected by a genetic disorder caused by mutations in one of about 30 genes responsible for monogenic podocytopathies [17,22]. However, a genetic etiology has been claimed for many SRNS patients classified as non-genetic by currently performed genetic testing.

Despite being usually recognized by standard diagnostic work-up because of clinical features suggestive of the syndromic picture, genetic nephropathies outside the podocytopathies spectrum (e.g., Alport syndrome, Dent disease) can rarely present as isolated SRNS or FSGS, preventing a precise diagnosis and leading to misclassification [74,75,76,77,78,79,80]. Indeed, genetic testing for podocytopathy genes is negative. According to the general concept, these conditions can be named phenocopies of monogenic podocytopathies, since they present with a clinical picture of apparently isolated SRNS but they are not caused by mutations in podocytes genes [81,82,83,84,85]. A comprehensive genetic testing in SRNS should therefore comprise phenocopy genes in order to provide a conclusive diagnosis and correctly classify disease entities (Table 2).

First insights into the importance of disease reclassification in SRNS came in recent years from case reports describing examples of phenocopies mimicking idiopathic SRNS that led to erroneous treatment. In 2013, Fervenza [76] reported the case of an 18-year-old patient presenting with nephrotic-range proteinuria who had been previously diagnosed as minimal change disease and then as “biopsy-missed” FSGS. Indeed, during the course of the disease, the patient received a therapeutic trial with steroids and cyclosporine. Only after treatment failure, further reconsideration of laboratory and biopsy findings allowed the suspicion of Dent disease that was then confirmed by genetic analysis, not performed earlier, showing a frame-shifting mutation in the *CLCN5* gene. A similar case was reported by Saida et al. [77].

Besides single case reports, the first systematic studies showing the role of mutations in other than common SRNS-causing genes, but clinically mimicking the phenotype, were performed in patients with mutations in the Alport syndrome-related gene COL4A [86]. Starting from the observation that patients with AD Alport syndrome due to heterozygous mutations in *COL4A3* and *COL4A4* could present with a variable clinical phenotype, including isolated severe proteinuria with FSGS at kidney biopsy, Malone et al. [81] speculated that some patients receiving a generic diagnosis of FSGS could instead be mutations carriers in *COL4A3* and *COL4A4* and so be misclassified. Using WES, they tested a cohort of 70 families with a diagnosis of familial FSGS. In seven out 70 families, genetic testing showed heterozygous mutations in *COL4A3* or *COL4A4* segregating with disease manifestations. Interestingly, in all seven families, there were individuals with nephrotic-range proteinuria and histologic features of FSGS by light microscopy. As the authors reported, these findings demonstrated a possible overlap between phenotypes induced by *COL4A3* and *COL4A4* variants and familial FSGS genes, suggesting that screening for rare variants/mutations in these genes in families referred with a diagnosis of familial FSGS is essential for better disease definition and treatment [81]. Similar results came from other studies where the genetic approach reclassified the diagnosis or the clinical suspicion of SRNS/FSGS as Alport syndrome spectrum [72,87,88,89]. Interestingly, some of these studies included also sporadic cases, demonstrating that the correct genetic diagnosis and disease reclassification are mandatory even in the absence of clear familial history [72]. In addition, attempts to identify subtle clinical findings related to the genetic diagnosis of Alport syndrome (e.g., glomerular basement membrane thinning/lamellation at electron microscopy) were sometimes performed, although not systematically [88,89]. A similar story applies to other genes, such as *PAX2* [79].

In the last few years, thanks to the recognition that many genetic mutations can mimic SRNS, therefore acting as phenocopies, the spectrum of genes responsible for SRNS has been considerably widened. In a recent paper, Warejko et al. aimed at detecting monogenic causes of early-onset SRNS in an international cohort of 300 families by using WES and analysis of a large panel of genes including “phenocopy genes”. The authors reported detection of phenocopies in almost 5% of patients with SRNS. The mutations were found in eight phenocopy genes, specifically *COL4A5*, *COL4A3*, *CLCN5*, *GLA*, *AGXT*, *CTNS*, *FN1* and *WDR19* [3]. Interestingly, the term “phenocopy” was used for the first time in this work in the field of SRNS. Following a similar approach, phenocopies of monogenic podocytopathies can actually be found in other studies assessing the role of NGS in nephropathies, either in children or adults [65,73,74,90,91,92]. In these studies, unbiased extended genetic testing allowed authors to turn the initial clinical diagnosis into a specific genetic diagnosis. Specifically, phenocopies of monogenic podocytopathies were detected at a rate of 1–5%. All these patients received a generic clinical diagnosis of SRNS or FSGS before undergoing genetic analysis [65,72,73,90,91,92].

From a clinical point of view, the latest progress in the diagnostic algorithm is the possibility to couple extended genetic analysis with reverse phenotyping, enabling a more precise differentiation between overlapping phenotypes and leading to a reclassification of the diagnosis in individual patients [4,90,93]. Indeed, including genes responsible for inherited nephropathies other than monogenic podocytopathies (ideally, all “CKD genes”) represents the first step for a correct diagnosis of SRNS. However, the occurrence of unexpected findings needs to be addressed in order to provide reliable diagnostic results (Figure 3B). In a recent paper, Landini et al. systematically addressed this issue [4]. The diagnostic algorithm coupled WES for an extended panel of 298 genes related to CKD (including, but not limited to, SRNS-related genes) and reverse phenotyping, namely the reevaluation of patients and their families after genetic testing, looking for previously overlooked clinical features of the underlying genetic diagnosis. This approach was applied to a cohort of 111 patients affected by early-onset NS, including 64 patients with SRNS. Of note, genetic testing included parents of probands, allowing for assessing the segregation of variants. According to the literature, disease-causing variants in podocytopathy genes were detected in 30% of patients. However, reverse phenotyping permitted identifying previously unrecognized clinical signs of an unexpected underlying genetic nephropathy mimicking SRNS in 18 out of 64 patients (28%), confirming the genetic diagnosis. Phenocopy genes identified were *COL4A3*, *COL4A4*, *COL4A5*, *LAMB2*, *GLA*, *FAT1*, *FAT4*, *PAX2*, *CLCN5*, *CTNS*, *LMX1B* and *KANK1* [4]. Very recently, clinical revaluation on the light of genetic findings was performed in selected cases of large cohorts of patients [90,91], confirming the utility of moving back from genotype to phenotype in order to reclassify diseases (Figure 3B). Interestingly, similar results have been provided also for other inherited kidney diseases, such as tubulopathies and nephronophthisis [68,69,94].

The high diagnostic capability to reclassify the original diagnosis based on clinical suspicion has also been recently confirmed in a real-life clinical setting by Jayasingh et al., who reported the analysis of the clinical impact of genomic testing in patients with suspected monogenic kidney diseases. In this work, NGS reclassified the clinical diagnosis with direct impact on subsequent clinical management and counseling [91].

## 5. Conclusions and Future Perspectives

The clinical and genetic heterogeneity of many inherited nephropathies is a challenging task.

However, the identification of the exact genetic cause of a disease is pivotal with regard to therapy, management and prognosis [95]. The promise of genomic medicine is to provide personalized care based on an individual’s genetic information [12]. The advent and spreading of NGS represented a milestone in this process [70,96]. However, understanding the relationship between genotype and phenotype for many forms of disease is complicated by locus heterogeneity, pleiotropy, incomplete penetrance and variable expressivity that can finally hamper our ability to pinpoint a causal etiology [13]. Idiopathic SRNS makes no exception.

The genetic basis of SRNS has been studied since the end of the 1990s, with the identification of mutations in *NPHS1* and *NPHS2* as responsible for monogenic forms of the disease. NGS markedly improved our understanding of the genetic mechanisms of SRNS, leading to the identification of more than 30 disease-causing genes. Although the high genetic heterogeneity can complicate the choice of optimal genetic testing modality (targeted panels, WES, WGS), the clinical utility of NGS in SRNS has been widely proven [3,23,24,25,26,27,28,29,30,31]. Indeed, it is common knowledge that about 30% of cases of early-onset SRNS are genetic in nature. They are referred to as monogenic podocytopathies. Times and costs are suitable for daily clinical practice so that genetic testing is frequently performed with the aim of confirming the diagnosis and investigating genotype–phenotype correlations. A further step in correctly identifying the genetic cause of SRNS, if present, as well as in understanding the role of the overall genetic background of each single patient and in elucidating potential genetic biomarkers of disease progression, is represented by the application of unbiased extended genetic testing (WES or WGS) including all the genes that can cause monogenic CKD, looking for phenocopies. The rationale for this strategy is based on the observation that patients with monogenic diseases such as Alport syndrome, Fabry disease, Dent disease or CAKUT can sometimes present atypically and go clinically unrecognized. In these cases, hypothesis-driven genetic testing would miss a correct diagnosis of the underlying disease. On the other hand, performing extended genetic testing in patients with SRNS compels clinicians to face the challenge of relying on unexpected genetic findings with the clinical picture. Reverse phenotyping represents one of the possible strategies to overcome this obstacle, leading to a personalized, genetic-based diagnosis, with integration of other “omics” (transcriptomics, proteomics, metabolomics) representing possible alternatives [12,95]. Of note, testing parents and/or first-degree family members is crucial in interpreting the results of genetic testing and for reverse phenotyping [95]. Besides ending the “diagnostic odyssey” and improving differential diagnosis by detecting phenocopies, this strategy is essential in initiating referral and evaluation for associated extra-renal features, tailoring therapies and referral to appropriate clinical trials when available (e.g., early onset of ACE-inhibitors for Alport syndrome), assessing prognosis and guiding family counseling (including donor selection for kidney transplantation). Finally, a molecular classification of SRNS can result in reconsidering the disease ontology, as occurs for other nephropathies [12].

As a consequence, in the near future, investigation for rare Mendelian disorders including SRNS will probably change radically. Indeed, much diagnostic genetic testing will move from the clinical genetics department and will be initiated by clinicians who, in turn, will be in charge of assessing the clinical significance of genetic findings, finally establishing a conclusive diagnosis. This approach, called “mainstreaming”, is a direct legacy of many NGS projects that led to the fundamentals of the “genomic era” in medicine [97]. However, integrating information coming from genetics is a delicate and critical process that has to be led by clinicians with solid and robust expertise in the field [98], in order to tailor the promise of personalized medicine to come in daily clinical practice, changing the way we take care of our patients.

## Figures and Tables

**Table 1:** Examples of phenocopies in human diseases. This is a table illustrating examples of phenocopies in different fields of medicine. For each disease, we report disease-causing genes (when known), phenocopies and corresponding mechanisms.

Disease (Phenotype)	Causative Gene/s	Phenocopy	Mechanism of Phenocopy Determination	Ref.
Pendred’s syndrome	*SLC26A4*	Endemic cretinism	Environmental	[34]
Monogenic skeletal disorders (e.g., Holt–Oram syndrome, radius aplasia-thrombocytopenia syndrome)	e.g., *TBX5*, *RBM8A*	Thalidomide embryopathy	Environmental	[34,38]
North Carolina macular dystrophy (NCMD) grade 3	locus chr5p15-p13	Congenital toxoplasmosis	Environmental	[41]
North Carolina macular dystrophy (NCMD) grade 1	*DHS6S1*	Foveal hypoplasia	Unknown	[41]
North Carolina macular dystrophy (NCMD) grade 2	*PROM1*	Torpedo maculopathy	Unknown	[41]
Hypertrophic cardiomyopathy	Sarcomeric genes	Glycogen storage diseases (e.g., Danon disease)	Monogenic	[42,43,44]
		Lisosomal storage diseases (e.g., Fabry disease)	Monogenic	
		Mitocondrial cytopathies (e.g., MELAS)	Monogenic	
		AL amyloidosis	Unknown	
Autoimmune lymphoproliferative syndrome (ALPS)	*FAS*, *FASLG*, *CASP10*	ALPS phenocopies	Somatic mutations in *FAS* (non-Mendelian genetic mechanism)	[47,48,49]
Cryopyrinopathies	*NLRP3*	Cryopyrinopathies phenocopies	Somatic mutations in *NLRP3* (non-Mendelian genetic mechanism)	[47]
Hereditary angioedema	*SERPING1*	Acquired angioedema	Autoantibodies anti-C1-inhibitor (complex mechanism)	[50]
Chronic mucocutaneous candidiasis	Genes encoding IL-17, IL-22	Recurrent fungal infections	Autoantibodies anti-IL17 or IL-22 (complex mechanism)	[51]
Familial Parkinson’s disease	*SNCA*, *LRRK2*, *PRKN*, *PINK1* and others	Familial Parkinson’s disease negative for mutations in known genes	Unknown	[53,54]
Familial breast cancer	*BRCA1*, *BRCA2*	Familial breast cancer negative for mutations in *BRCA* genes	Unknown (probably genetic)	[56,57]
Huntington disease (HD)	*HTT*	HD phenocopies	Monogenic (mutations in genes different from *HTT*)	[58,59]
Brugada syndrome	*SCN5A*	Brugada phenocopies (e.g., metabolic abnormalities, ischemia, mechanic compression, myocardial and pericardial diseases)	Unknown (probably complex)	[60,61,62]

**Table 2:** Phenocopy genes. This is a table illustrating phenocopy genes and the disease usually caused by their mutations (phenotype).

Gene		Phenotype	Ref.
*CLCN5*	CHLORIDE CHANNEL 5	Dent disease	[3,4,76,77,91]
*COL4A3*	COLLAGEN, TYPE IV, ALPHA-3	Alport syndrome	[3,4,72,81,88,89,90]
*COL4A4*	COLLAGEN, TYPE IV, ALPHA-4	Alport syndrome	[72,74,81,89]
*COL4A5*	COLLAGEN, TYPE IV, ALPHA-5	Alport syndrome	[3,72,89,90]
*GLA*	GALACTOSIDASE, ALPHA	Fabry disease	[3,4,90]
*AGXT*	ALANINE-GLYOXYLATE AMINOTRANSFERASE	Hyperoxaluria, primary, type 1	[3]
*CTNS*	CYSTINOSIN	Cystinosis	[3,4]
*FN1*	FIBRONECTIN 1	Glomerulopathy with fibronectin deposits 2	[3]
*WDR19*	WD REPEAT-CONTAINING PROTEIN 19	Nephronophthisis 13	[3]
*LAMB2*	LAMININ, BETA-2	Pierson syndrome	[4]
*FAT1*	FAT ATYPICAL CADHERIN 1	FAT1-related glomerulo-tubular nephropathy	[4]
*FAT4*	FAT ATYPICAL CADHERIN 4	Van Maldergem syndrome 2	[4]
*PAX2*	PAIRED BOX GENE 2	Papillo-renal syndrome/FSGS	[4]
*LMX1B*	LIM HOMEOBOX TRANSCRIPTION FACTOR 1, BETA	Nail–patella syndrome	[4]
*KANK1*	KN MOTIF- AND ANKYRIN REPEAT DOMAIN-CONTAINING PROTEIN 1	Cerebral palsy	[4]
